# Supplementary material for: Light-triggered multi-joint microactuator fabricated by two-in-one femtosecond laser writing
Source: Nat Commun. 2023 Jul 17;14:4273. doi: 10.1038/s41467-023-40038-x (PMC10352372; doi:10.1038/s41467-023-40038-x)
Supplement: Supplementary file 1 — Supplementary Information [file 41467_2023_40038_MOESM1_ESM.pdf]

Supplementary Information for

Light-triggered multi-joint microactuator fabricated by two-in-one femtosecond laser writing

Chen Xin[1, 2, †], Zhongguo Ren[1, †], Leran Zhang[1], Liang Yang[3], Dawei Wang[1], Yanlei Hu[1], Jiawen Li[1], Jiaru Chu[1], Li Zhang[2], and Dong Wu[1]*

[1]Key Laboratory of Precision Scientific Instrumentation of Anhui Higher Education Institutes, CAS Key Laboratory of Mechanical Behavior and Design of Materials, Department of Precision Machinery and Precision Instrumentation, University of Science and Technology of China, Hefei 230026, China.
[2]Department of Mechanical and Automation Engineering, The Chinese University of Hong Kong, Hong Kong 999077, China
[3]Suzhou Institute for Advanced Research, University of Science and Technology of China, Minde Building, Renai Road, 215123 Suzhou, P. R. China
[†]These authors contributed equally to this work.

*Corresponding author. Email: dongwu@ustc.edu.cn

This PDF file includes:

Supplementary Note 1 to Note 5
Supplementary Fig. 1 to Fig. 24
Supplementary Table 1

Supplementary Note 1 Controllable multi foci number, distribution and spacing

To precisely control the distance between the multifocal points, we investigated the mathematical relationship between the distance between double foci at the focal plane of high numerical aperture objectives and the pixel distance between two foci on the design target image. The spatial light modulator can be regarded as a two-dimensional grating structure composed of many pixels. The image formed by the two-dimensional grating on the focal plane of the lens L_1 passes through the lens L_2 and the high numerical aperture objective lens and is imaged on the focal plane of the objective lens (Supplementary Fig. 3). According to the multi-slit diffraction theory, the distance between two foci corresponding to the focal plane of the objective lens can be obtained

$$\Delta L = \frac{\lambda f_1}{d_{pixel} N_{pixel}} \frac{f_{objective}}{f_2} \quad (S1)$$

where λ is the wavelength of the femtosecond laser, f_1 and f_2 are the focal lengths of lens L_1 and lens L_2 respectively, $f_{objective}$ is the focal length of the objective lens, d_{pixel} is the pixel period of the LCoS SLM liquid crystal panel, N pixel is the number of pixels in the short axis direction of the LCoS SLM, according to the formula, the real distance corresponding to a pixel on the design drawing on the focal plane of the high numerical aperture objective lens can be calculated. λ in our experiments is 800 nm, f_1 and f_2 are 600 mm and 200 mm, respectively, objective is 9 mm, dpixel is 8 µm, and N_{pixel} is 1080. According to the formula, it can be calculated that when the distance between the two focal points on the design drawing is 1 pixel, the distance between the two foci imaged on the focal plane of the objective lens is 2.5 µm, so we can design Accurately calculate the distance between multifocal points.

Supplementary Note 2 Generation of muti-focal hologram in 3D space

As shown in Supplementary Fig. 3, the holographic multifocal beam is generated by the optical setup. Loading a computer-generated hologram (CGH) on the SLM, the desired multifocal pattern can be reconstructed in the focal region of the objective and used for multi-joint actuation. The CGH is first obtained from a pre-designed pixelated target pattern by the Gerchberg-Saxton (GS) algorithm to generate a phase depth pattern. To avoid the interference of 0-order light, we will superimpose a blazed grating (BG) phase on the initial phase. Finally, the phase distribution of the CGH displayed on the SLM is expressed as,

$$Ph_1(x,y) = mod(\varphi_{initial}(x,y) + \varphi_{BG}(x,y), 2\pi) \quad (S2)$$

where (x, y) are Cartesian coordinates, φ origin represents the initial multifocal phase calculated by the G algorithm, and φ_{BG} represents the grating (BG) phase that separates the modulated first diffraction order from the zeroth order beam.

The above is the production method of the two-dimensional multifocal light field in the XY plane of Z=0 µm, and the adjustable multifocal in Z axis needs to add a lens phase to the original hologram, which can be calculated as,

$$\varphi_{lens}(x,y) = \frac{2\pi \Delta f (x^2 + y^2)}{\lambda f^2} \quad (S3)$$

where Δf and f are the change in focal length and the equivalent focal length. Thus, final phase of focal point with adjustable position is

$$Ph_2(x,y) = mod(\varphi_{initial}(x,y) + \varphi_{lens}(x,y) + \varphi_{BG}(x,y), 2\pi) \qquad (S4)$$

For multiple focal phases on different planes, it can be expressed as the vector superposition of the respective phases, and it can be expressed as,

$$Ph_{3D}(x,y) = mod\ (\varphi_1(x,y) + \varphi_2(x,y), 2\pi) \qquad (S5)$$

where $\varphi_1(x,y)$ and $\varphi_1(x,y)$ are phase of point 1 in Z=0 μm and point 2 in Z =15 μm.

Under the same energy of incident light, as the number of focal points increases, the energy distributed by each focal point decreases gradually. Therefore, we superimpose an attenuation factor (q) on the holographic phase of different foci numbers to ensure that when the beam switches to different foci numbers, each foci distributes approximately the same energy density. Finally, the phase hologram of a multifocal beam is expressed as,

$$Ph(x,y) = q(Ph_{3D}) \qquad (S6)$$

where the q values corresponding to single foci, double foci, four foci, and eight foci are 1, 0.8, 0.6, and 0.4 respectively.

Supplementary Note 3 Simulation of multi-foci beam by Debye diffraction theory

In our study, the incidence in our experiments is assumed to be Gaussian beam with plane wave front. Thus, the reflective light of the Gaussian beam after SLM modulation can be describe as:

$$E(\rho,\varphi,z) = -\frac{exp(ikz)}{ikz} exp\left(\frac{ik\rho^2}{2z}\right) \int_0^R \int_0^{2\pi} exp(il\theta)\, exp\left(\frac{ikr^2}{2z}\right) exp\left[\frac{-ikr\rho\, cos(\theta-\varphi)}{z}\right] 2\pi r dr d\theta \ (S7)$$

Where ρ and φ are polar coordinates in the observation plane. l is the topological charge, and R is the radius of the CGH. For direct laser writing of hollow microhelices, the optical vortex needs to be tightly focused with an oil immersed objective (NA=1.35). We calculate the intensity distribution in the focal plane by the Debye diffraction theory, which is different from the scalar diffraction theory. The theory describes the depolarization effect of a high NA objective due to the paraxial approximation does not consider the vectorial nature. Therefore, the three orthogonal field components Ex, Ey, and Ez are calculated. The optical intensities near the focal spot are derived as:

$$E(x_2,y_2,z_2) = -\frac{iC}{\lambda}\int_0^{\alpha}\int_0^{2\pi} sin\,\theta E_{obj}(\theta,\varphi)\sqrt{cos\,\theta}\,P(\theta,\varphi) \times exp[ikn(z_2\, cos\,\theta + x_2\, sin\,\theta\, cos\,\varphi + y_2\, sin\,\theta\, sin\,\varphi)]d\theta d\varphi \qquad (S8)$$

Where C is a constant, $E_{obj}(\theta,\varphi)$ is the electric field at the entrance pupil of the objective, $k = 2\pi/\lambda$ is the wave number, which the wavelength of incident light is 800 nm. n is the refractive index of the immersion medium, θ represents the focusing angel of the objective lens, and φ is the azimuthal angle of the object plane. α is the maximum focusing angle of the objective lens and can be expressed by the formula:

$$\alpha = arc\,cos\left(\frac{NA}{n}\right) \tag{S9}$$

where NA=0.45 is numerical aperture of objective lens. *P (θ, φ)* indicates the polarization state of the EM field in the focal region, which can be rewritten as:

$$P(\theta,\varphi) = [1 + (cos\,\theta - 1)cos^2\varphi]\boldsymbol{i} + [(cos\,\theta - 1)\,cos\,\varphi\,sin\,\varphi]\boldsymbol{j} - (sin\,\theta\,cos\,\varphi)\boldsymbol{k} \tag{S10}$$

for incidence with linear polarization at X direction, as used in our experiment.

Supplementary Note 4 Simulation of the microactuator deformation

The numerical simulations are performed in the commercial finite-element software COMSOL. The thermomechanical analysis of the microrobots is executed in the Solid Mechanics module of COMSOL. Because of the larger shrinking ratio of the lower-crosslinking layer than the higher-crosslinking layer, the elastic bending of the heated joints is modeled using the anisotropic thermal strain across their thickness. The parameters used to model the two distinct layers of materials are chosen in such way that the numerical results closely resembled the experimental situations in the simplest circular plate cases. In this way, we obtained a thermal shrinking ratio α_1=0.35 and α_2=0.06 for the circular plates with lower-crosslinking density and higher-crosslinking density, respectively. All other more complex microstructure calculations are performed with this set of parameters. For the heat diffusion calculations, different microrobots with a central base immersed in sufficiently large water are chosen. The incident laser beam with Gaussian distribution is applied on the surface of the joints of microrobots, and the outer water bath is set to room temperature. For the deformation calculations, the response of the microrobots to changes in the temperature of the joints is introduced as volumetric stress.

Supplementary Note 5 Mechanical test and calculation of the hydrogel

According to the reviewer's advice, we added a series of experiments to refine the manuscript. Firstly, we test the mechanical property (Young's modulus (E_0)) of hydrogels with different components by a Micromechanical Testing System (FemtoTools, FT-MTA02). An FT-S100000 microforce sensing probe with a force range of ±100,000 µN and a resolution of 5 µN is used with a 50 µm × 50 µm tip to measure the compression forces of blocks of polymerized hydrogel with dimensions of 40 µm × 40 µm × 15 µm (Supplementary Fig. 7a). Hydrogels (with and without PVP) are printed with the same laser scanning parameters (PLP=30 mW, ST= 3ms). Subsequently, the pressure vs displacement curves of the hydrogels are measured (Supplementary Fig. 7b). The E_0 could be calculated as,

$$E_0 = P/\varepsilon = \frac{F/_S}{\Delta L/_L} \tag{S11}$$

Where, S=1.6×10^{-9} m^2, L=1.5×10^{-5} m, ΔL=10^{-6} m are the area, height, and compression displacement of the microplate. F is the pressure loaded on the microplate (327 µN and 676 µN of hydrogels with and without PVP addition).

Thus, compared with the hydrogel microstructure without PVP addition (3.07 Mpa), the hydrogel microstructure with PVP addition (5% wt) has a higher E_0 of 6.34 Mpa. It was also evident that processing microstructures in hydrogels without PVP addition led to significant defects during processing. On the contrary, the PVP-added hydrogel can print high-quality 3D microactuators

(Supplementary Fig. 7b). In addition, we test three groups of hydrogels (with PVP) with different laser scanning times (1 ms, 2 ms, and 3 ms), which have F of 165 μN, 351 μN, and 676 μN, resulting in E_0 of 1.55 Mpa, 3.29 Mpa, and 6.34 Mpa, respectively (Supplementary Fig. 11b)).

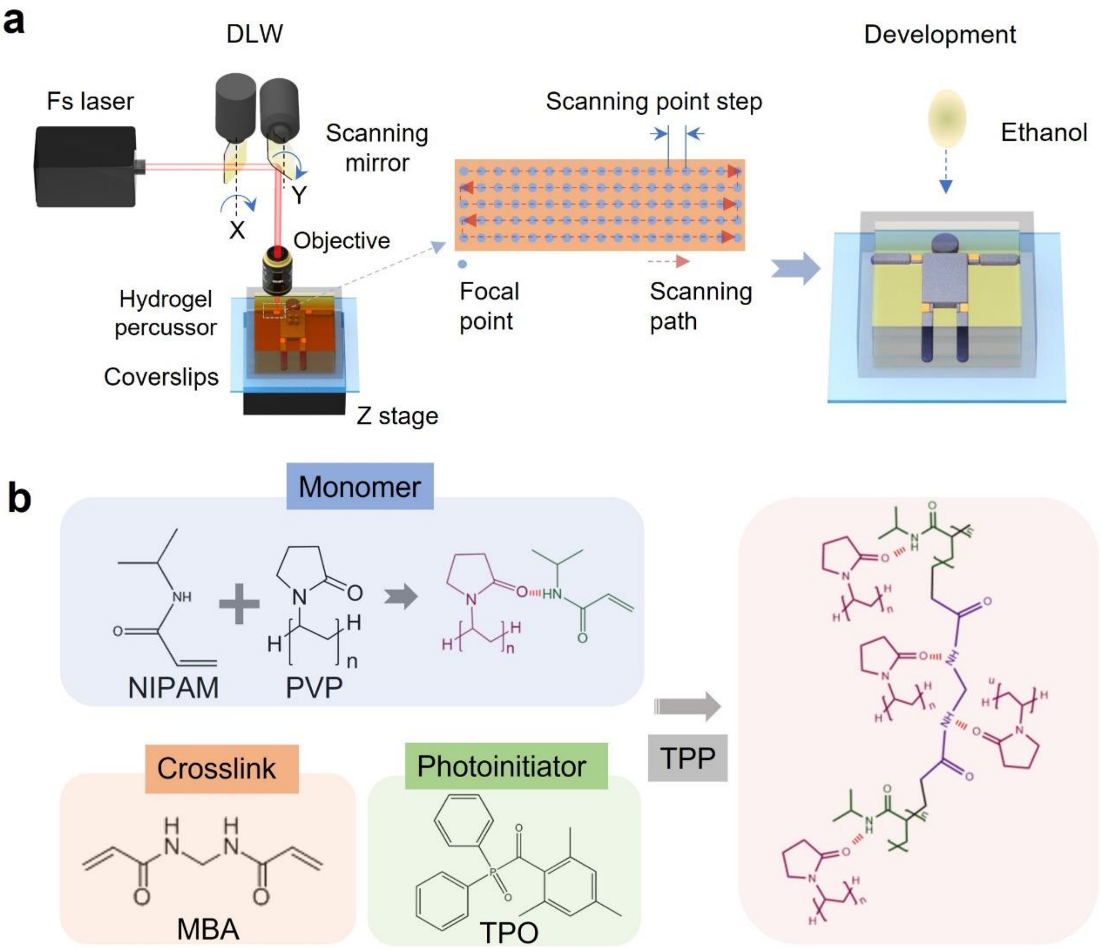

Supplementary Fig. 1 Direct laser writing (DLW) system with the replaceable liquid tank for hydrogel and silver nanoparticles (Ag NPs) fabrication. (**a**) The femtosecond (fs) laser is focused through a high numerical aperture objective lens and then scanned point-to-point to fabricate multi-joint microactuator (MJMA). (**b**) Components of hydrogels, including polymerizable monomers (N-Isopropyl acrylamide, NIPAM), crosslinkers (Methylene-Bis-Acrylamide, MBA), and photoinitiators (Diphenyl (2,4,6-trimethyl benzoyl) phosphine oxide, TPO). When the hydrogel is polymerized, hydrogen bonding occurs between NIPAM and polyvinylpyrrolidone (PVP) to enhance the hydrogel's mechanical property. TPP represents two-photon polymerization.

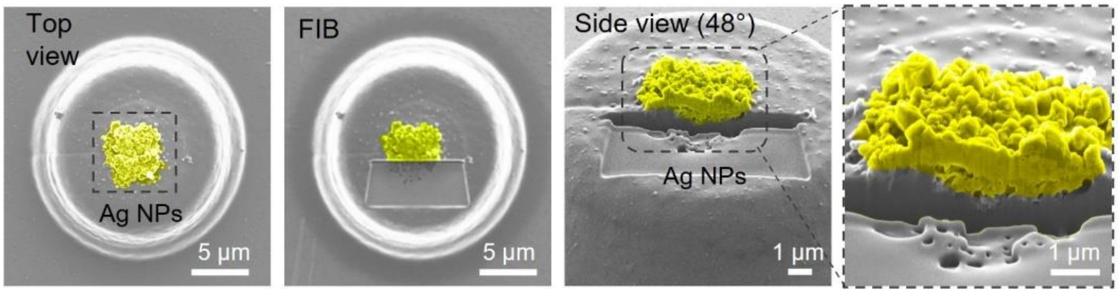

Supplementary Fig. 2 After focused ion beam (FIB) cutting, Ag NPs (yellow) can be seen deposited on the surface of the hydrogel in the side view (48°). Ag NPs represents silver nanoparticles.

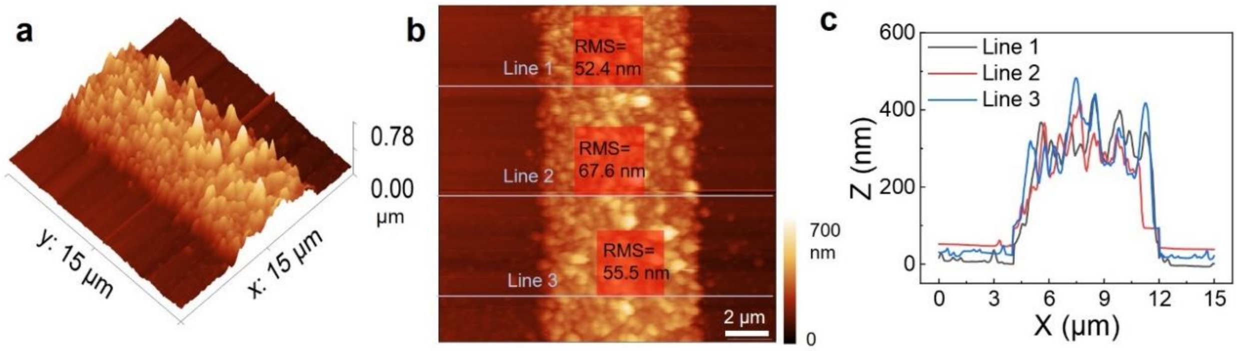

Supplementary Fig. 3 Characterization of thickness and roughness of Ag NPs layer. (**a**) AFM scanned a 3D image of the Ag NPs layer. (**b**) The roughness of the three areas of the Ag NPs layer are 52.4 nm, 67.6 nm, and 55.5 nm, respectively. RMS represents root mean square value of roughness. (**c**) The thickness of the Ag NPs layer on the three extraction lines is between 300 nm and 400 nm.

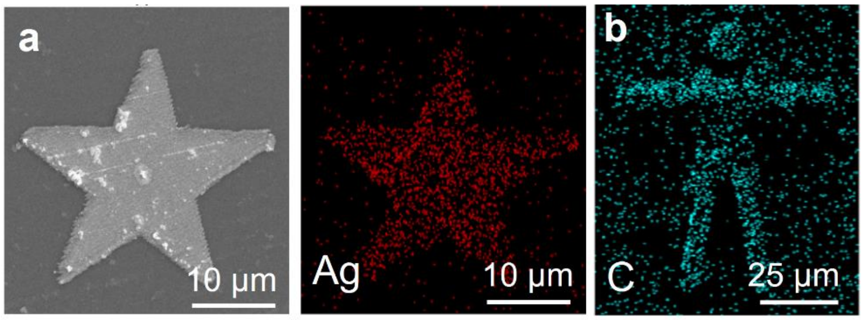

Supplementary Fig. 4 (**a**) Scanning electron microscope (SEM) and Energy Dispersive Spectrometer (EDS) images of a pentagram composed of photo-reduced Ag NPs, where the red color represents the area where the Ag NPs accumulate. (**b**) EDS image of humanoid MJMA, where the blue color represents the area where the hydrogel aggregates. Ag and C represent silver and carbon elements, respectively.

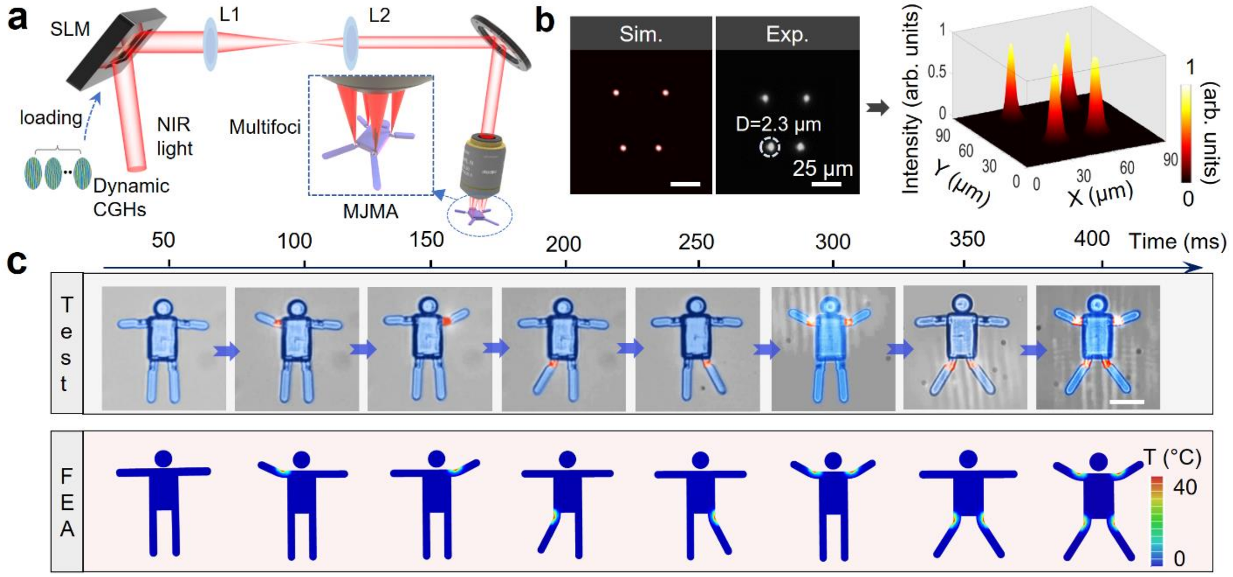

Supplementary Fig. 5 (**a**) Reconfigurable humanoid MJMA is controllably triggered by dynamic multi foci light fields. (**b**) Four foci are modulated by the Gerchberg-Saxton (GS) algorithm to control four joints deformation, where the spatial (3D) position and intensity of the focal points can be flexibly adjusted. Sim. and Exp. represent the simulation and experiment results of multi-foci distribution. (**c**) Experimental tests and finite element analysis of a micro-humanoid robot that can reconstruct eight different morphologies by different light foci stimulation. Simulation results showing the temperature of the micro joints when stimulated by light exceeds the critical value of hydrogel deformation temperature (32 °C). Scale bar, (c) 20 μm.

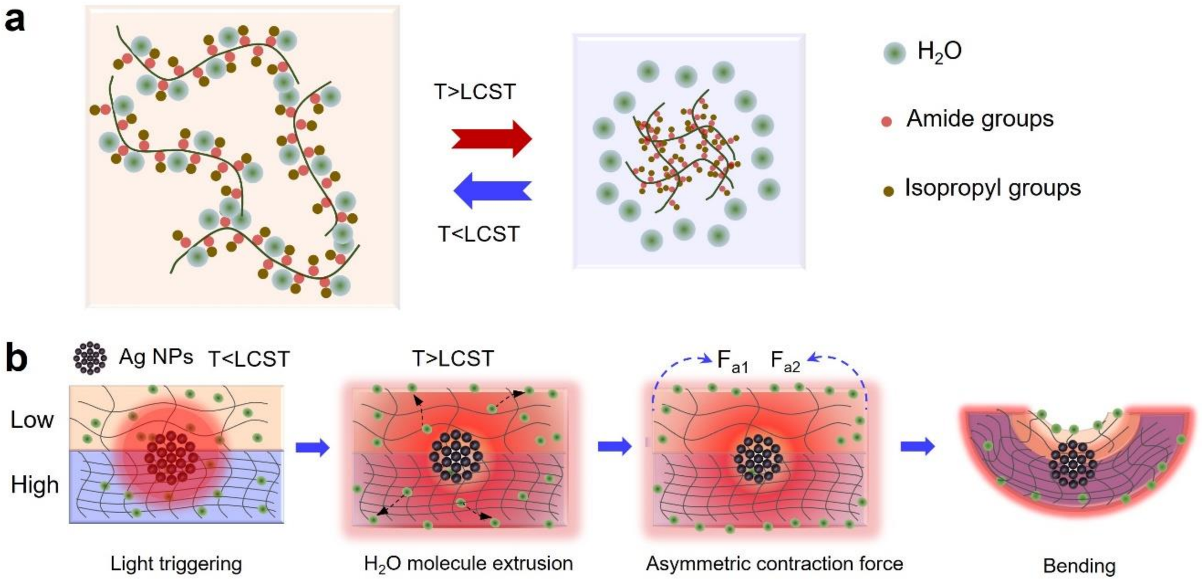

Supplementary Fig. 6 (**a**) The mechanism of hydrogel response to temperature, when the temperature (T) > low critical solution temperature (LCST), the hydrogel shrinks, otherwise it swells. (**b**) The process of hydrogel joint flexion. First, light radiation of Ag NPs generates a large amount of heat, which is subsequently transferred to the hydrogel, causing the temperature of the hydrogel to reach LCST. At this point, the hydrogel exposes hydrophobic groups to expel water molecules to produce a contraction force. The hydrogel part with low cross-link density will generate bigger contraction force (F_{a1} and F_{a2}), which will eventually lead to the bending of the hydrogel joints.

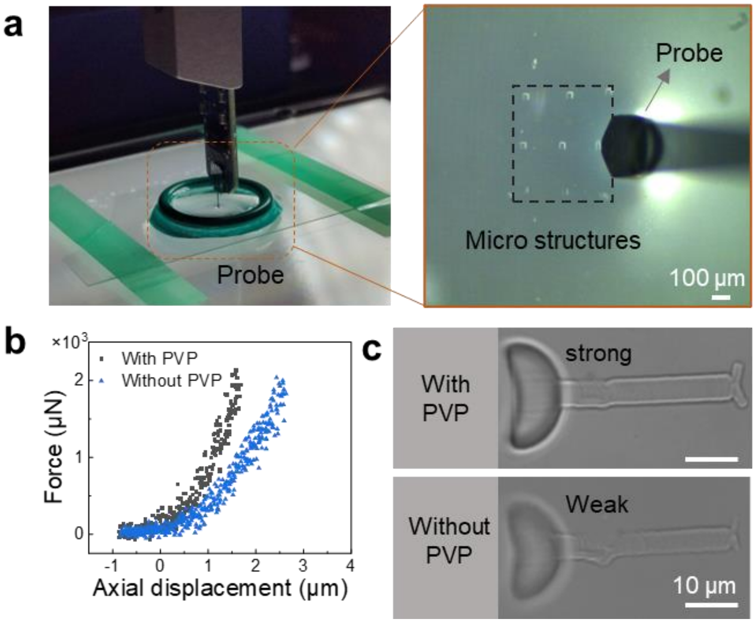

Supplementary Fig. 7 Mechanical test of hydrogels with different components. (a) Setup of a micromechanical testing system. (b) Mechanical test of hydrogels with and without polyvinylpyrrolidone (PVP) additions. (c) Compared to hydrogel without PVP, hydrogel with PVP demonstrates stronger structural properties.

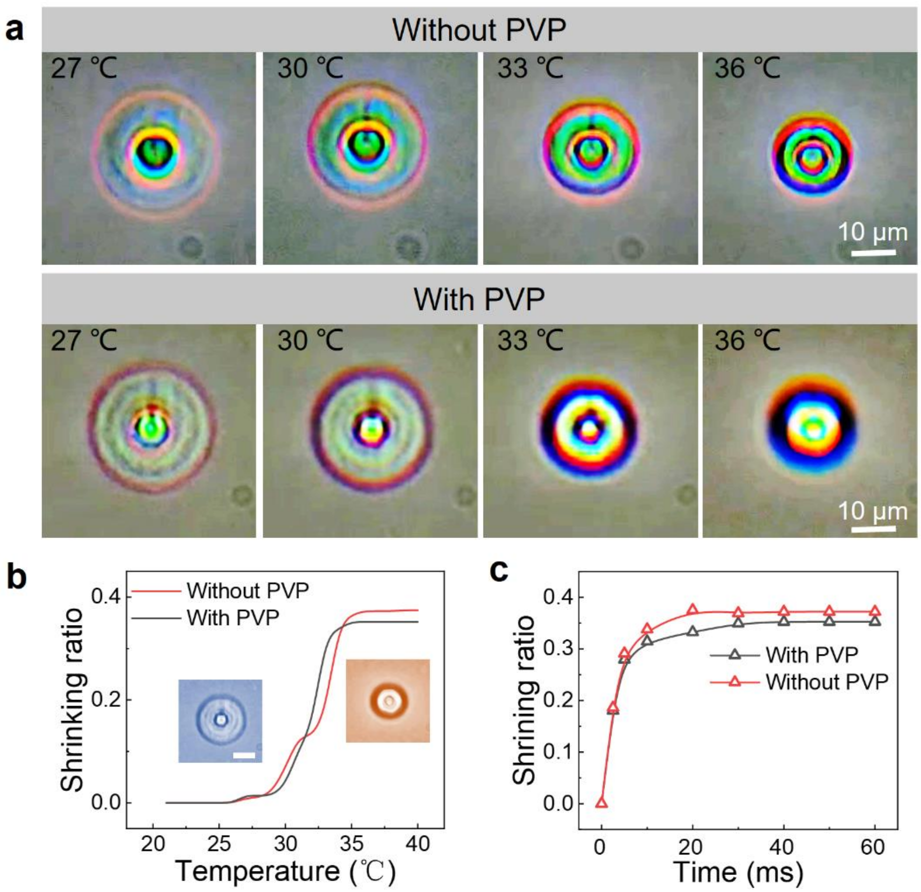

Supplementary Fig. 8 Effect of polyvinylpyrrolidone (PVP) on the thermal sensitivity of hydrogels. (**a**) Time-lapse images of shrinkage of hydrogel microplates with and without PVP addition with increasing temperature. (**b**) The thermal response characteristics of hydrogel microstructures before and after the addition of PVP. (**c**) Photothermal response properties of hydrogels with PVP addition, where shrinkage and response times are comparable to those of hydrogels without PVP addition. Scale bars, (**b**) 10 μm.

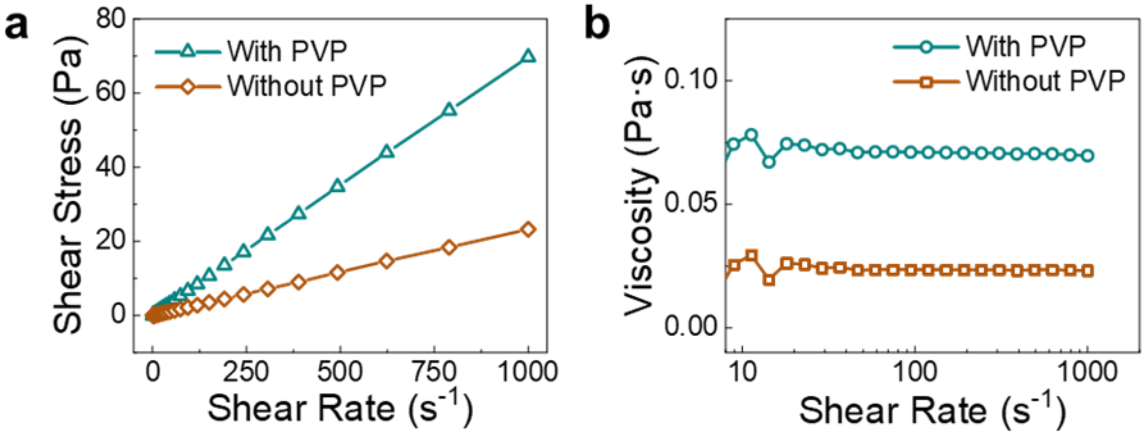

Supplementary Fig. 9 Rheology and viscosity testing of prepared hydrogels (All experiments are tested under 25 °C). (**a**) The relationship between shear stress and shear rate of hydrogels with and without polyvinylpyrrolidone (PVP) addition. (**b**) The viscosity of hydrogel precursor with PVP addition is 0.073 Pa·s, higher than that of hydrogel without PVP addition (0.024 Pa·s).

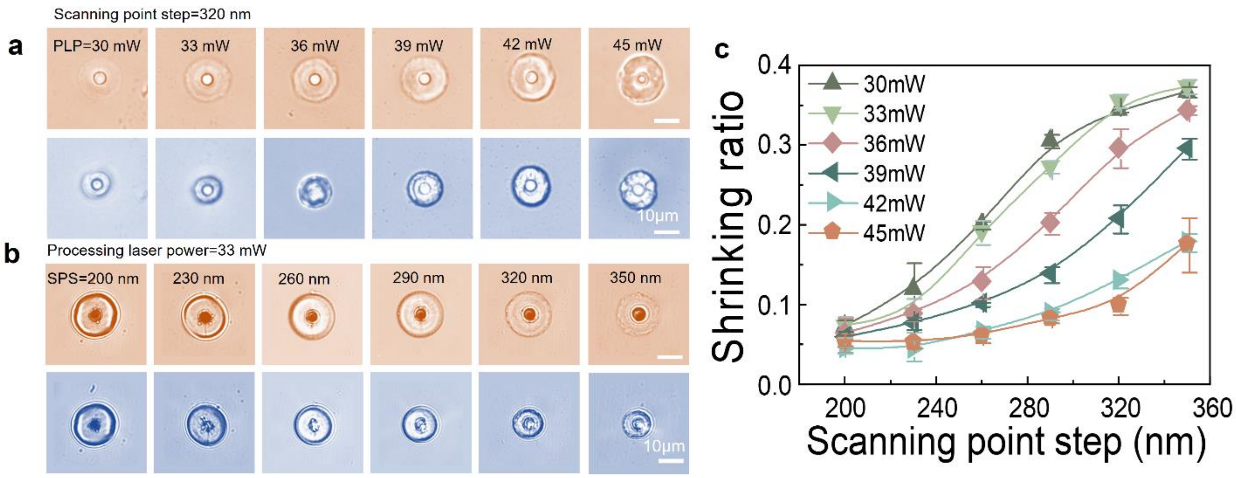

Supplementary Fig. 10 (**a**) Microscopic images of shrinking microplates processed with different processing laser powers (processing laser power (PLP) from 30 mW to 45 mW). The complete structure cannot be processed when the PLP is lower than 30 mW and an optical micro-explosion will be produced, when the PLP is higher than 45 mW. (**b**) Microscopic images of the initial and heating shrinkage of microplates are processed with different scanning point step (Scanning point step (SPS) from 200 nm to 350 nm). The microstructure has poor mechanical properties when SPS > 350 nm. (**c**) Quantity relationship between shrinkage ratio and scanning powers and scanning point step length. Considering the shrinkage rate and the ability to complete the processing structure, PLP (33 mW) and SPS (320 nm) are the main processing parameters adopted in our study. Error bars stand for the standard error (n = 3).

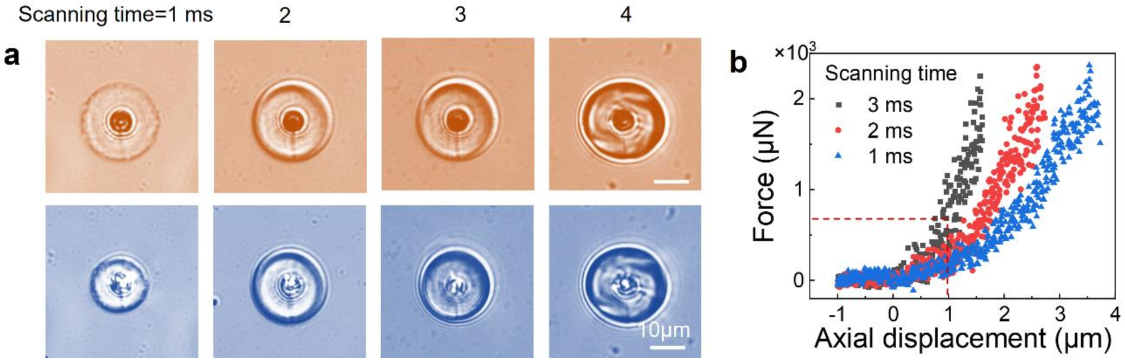

Supplementary Fig. 11 The microscopic images of the microplate with different scanning time (ST). (**a**) When ST increases, the shrinkage ratio of the microplate decreases. (**b**) Mechanical test of hydrogels (with polyvinylpyrrolidone (PVP)) with different laser scanning times (1~3 ms).

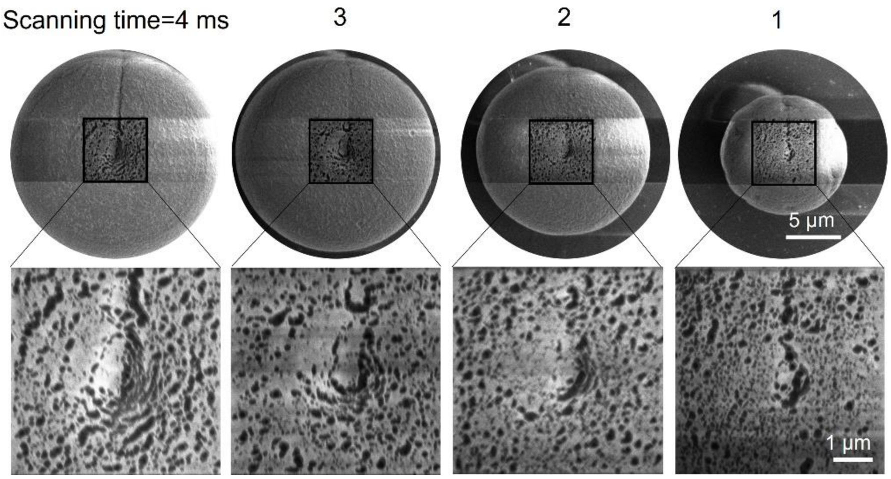

Supplementary Fig. 12 Scanning electron microscope image of the pores inside the hydrogel, the longer the scanning time (ST), the bigger the porosity retained by the structure and the smaller the volume shrinkage. After 30 s of focused ion beam (FIB) treatment, the nanopores inside the hydrogel are observed.

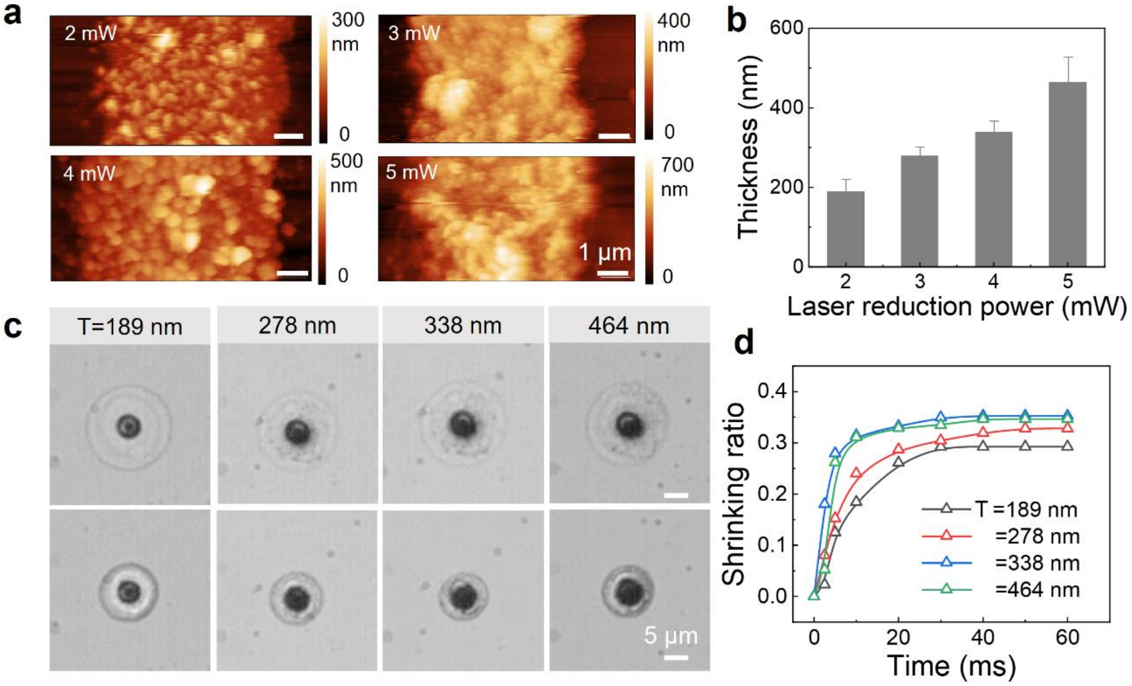

Supplementary Fig. 13 The effects of the Ag NPs thickness on the photothermal effect. (**a**) AFM images of Ag NPs layers fabricated with different laser reduction powers. (**b**) The layer thickness of Ag NPs increases from 189 nm to 464 nm with the increase of laser reduction power (2 mW~5 mW). (**c**) Photothermal shrinkage microscope images of microplates with different thicknesses (T) of Ag NPs layer. (d) When the layer thickness of Ag NPs is less than 200 nm, the shrinkage of the microplate is only 0.29. When the layer thickness of Ag NPs is higher than 300 nm, the shrinkage rate of hydrogel reaches the maximum value (~0.35). Error bars stand for the standard error (n = 3).

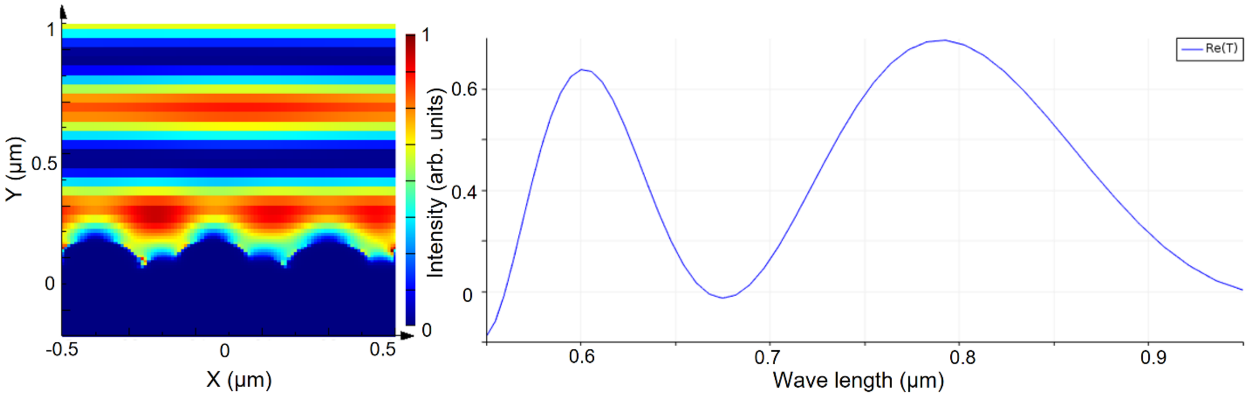

Supplementary Fig. 14 When the excitation light wavelength is near 800 nm, the stacked nanoparticle surface generates a strong electric field enhancement, thus producing a strong photothermal conversion effect. Color bar represents electric field intensity.

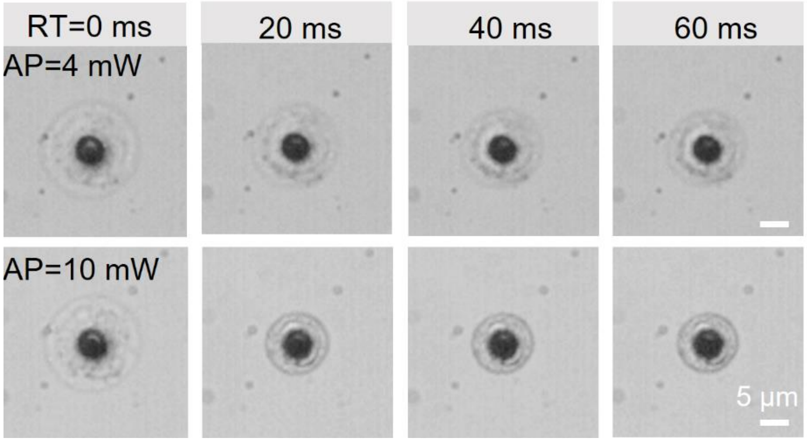

Supplementary Fig. 15 Time-lapse images (0~60 ms) of microplates stimulated by light under high-speed camera (2000 fps). The shrinkage of the microplate can completely increase to the maximum within 60 ms when AP is 10 mW. RT and AP represent the response time and actuation power, respectively.

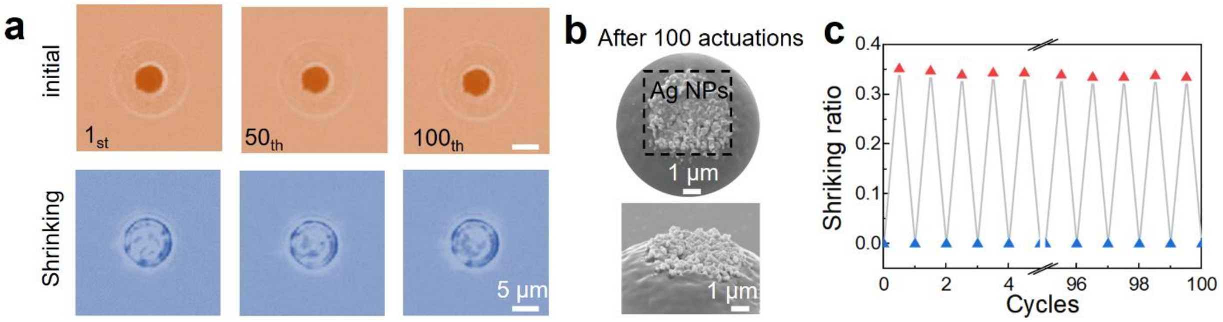

Supplementary Fig. 16 The cycle deformation test of the microplate. (**a**) The microscopic images of the microplate in expanding and shrinking state in the 1st, 50th, and 100th cycle. (**b**) The SEM image of Ag NPs layer deposited on the surface of hydrogel after 100 times actuation. (**c**) The microplate exhibits stable deformation under 100 cycles.

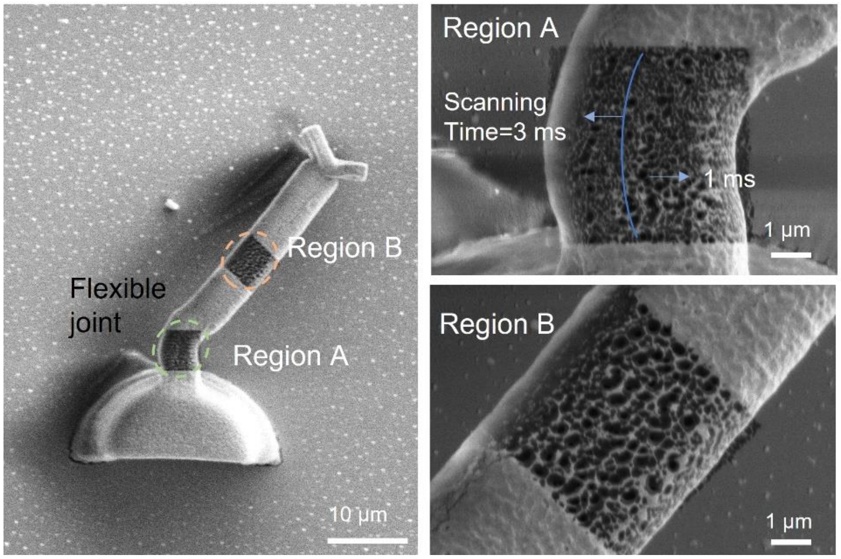

Supplementary Fig. 17 The porosity of different parts of the micro robotic arm, the porosity at the hinge is significantly different (region A), and the other parts are uniform (region B). After 30 s of focused ion beam (FIB) treatment, the nanopores inside the hydrogel are observed.

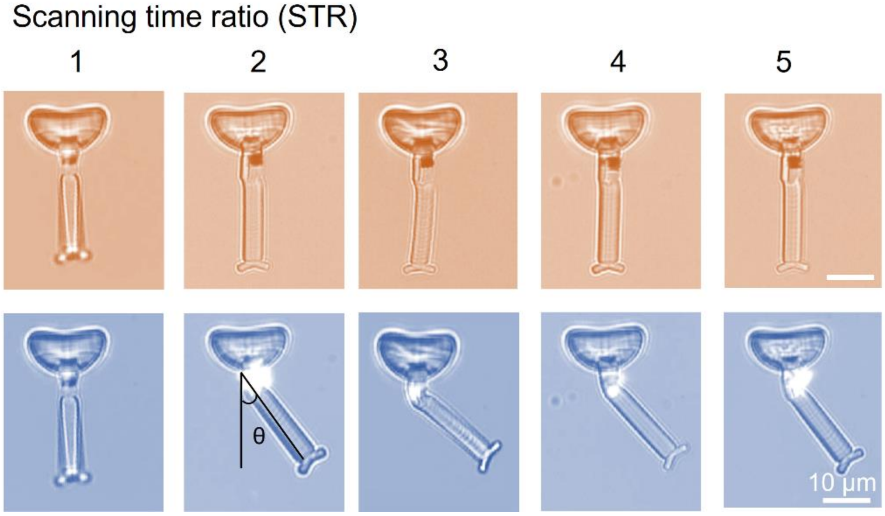

Supplementary Fig. 18 The microscopic images of the micro robotic arm (MRA) with different point scanning time ratios (STRs). The bending angle (θ) of the MRA first increases and then decreases with the increase of STR. The initial increase in the bending angle is because increasing the ST on one side reduces the shrinkage rate of this part, resulting in an increase in the bending angle. However, as the STR continues to increase (STR > 3), the degree of cross-linking is too high, resulting in a decrease in the bending angle.

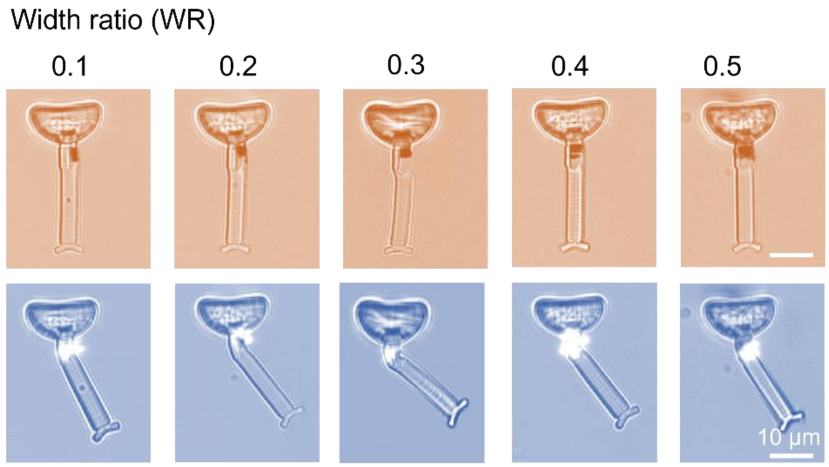

Supplementary Fig. 19 The microscopic images of MRA with different scanning width ratios (WRs). The bending angle of the MRA first increases and then decreases as the scan WR increases.

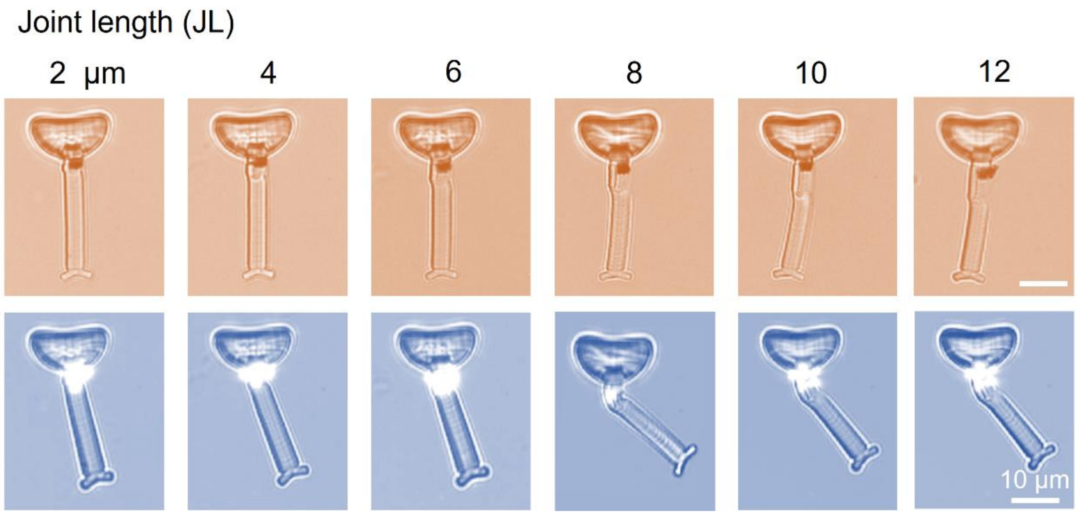

Supplementary Fig. 20 The microscopic images of MRA with different joint lengths (JLs). As the JL increases, the bending angle of the MRA first increases and then remains stable.

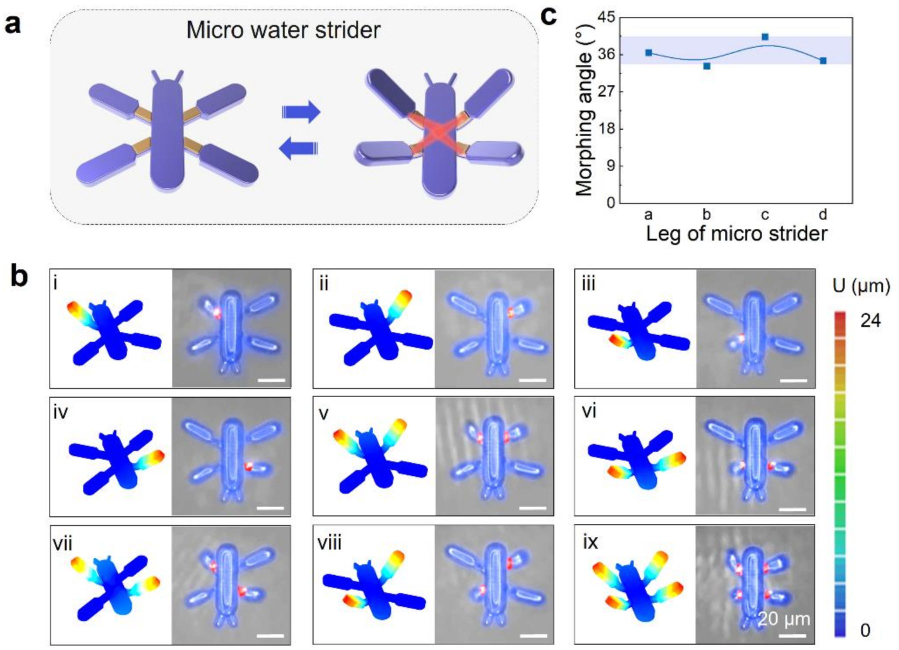

Supplementary Fig. 21 (**a**) The schematic diagram of the morphing of a micro water strider stimulated by light heating. (**b**) Light-triggered micro water strider has eight morphologies (modes i to ix). Finite element simulation results accurately predict the degree of deformation of the legs of a light-triggered water strider. U represents the displacement the actuator. (**c**) Light-triggered deformation of the four legs of a micro water strider with ultra-high consistency.

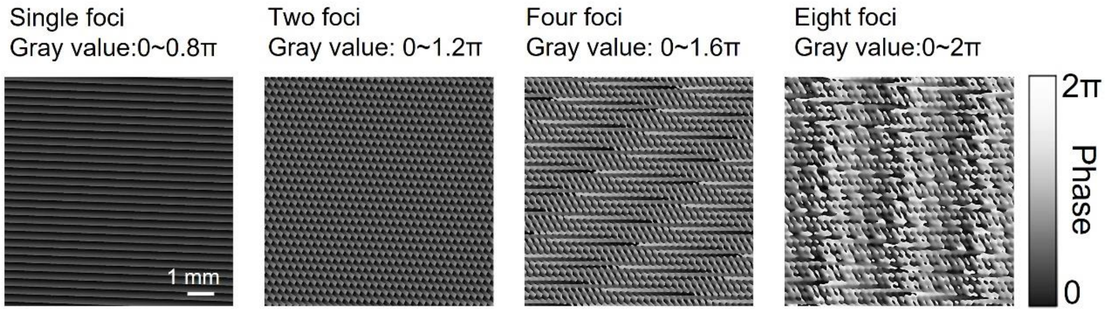

Supplementary Fig. 22 As the number of focal points increases, the energy density of each focal point decreases. Therefore, it is necessary to superimpose a grayscale attenuation factor on the holograms with different focal point numbers. The grayscale attenuation factors corresponding to single foci, double foci, four foci, and eight foci holograms are 0.4, 0.6, 0.8, and 1 respectively. All of the holograms were calculated by Gerchberg-Saxton (GS) algorithm using MATLAB 2021a. Each pixel point on the hologram represents 8 μm on the spatial light modulator.

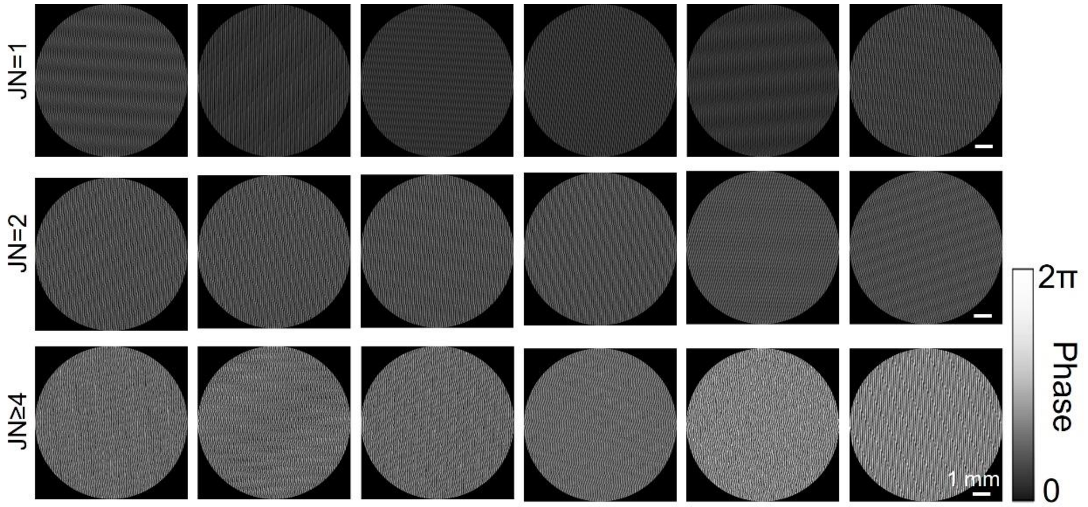

Supplementary Fig. 23 The holograms corresponding to single-joint, double-joints and multi-joint deformations are realized with a humanoid MJMA. By adjusting the overall gray level of the hologram to control the energy density of different numbers of focal points, preventing the energy from changing dramatically during the beam switching process. All of the holograms were calculated by the Gerchberg-Saxton (GS) algorithm using MATLAB 2021a. Each pixel point on the hologram represents 8 μm on the spatial light modulator. JN is denoted as joints number.

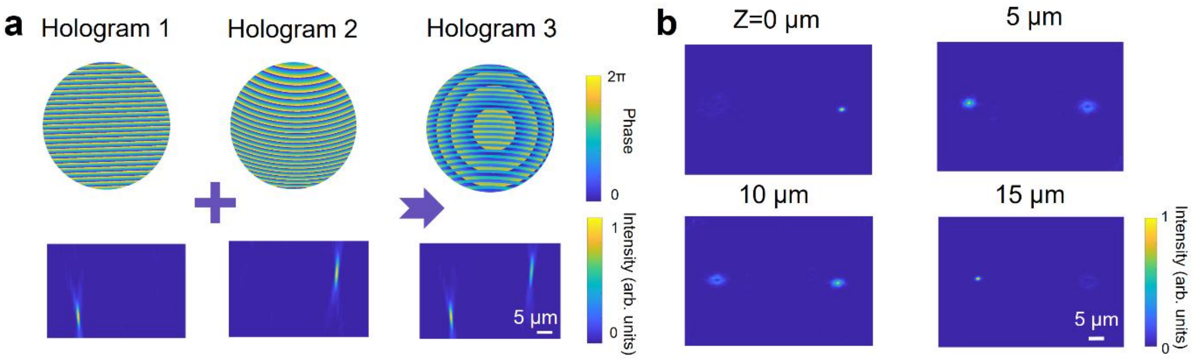

Supplementary Fig. 24 (**a**) Superposition of holograms of single foci at different Z depths can spatially generate bifocals. (**b**) Simulation of light intensity distribution of double focal points at different Z depths in 3D space without any crosstalk between the double foci.

Soft actuators	*Modality numbers	Size	Structure	Respose time	Response Materials	Stimuli mode
Ref. [1]	2	>1 cm	3D	>1 min	Hydrogel	Water
Ref. [2]	2	>2 mm	2D	>1 min	Hydrogel	pH
Ref. [3]	2	~100 µm	3D	NA	Hydrogel	pH
Ref. [4]	2	>1 mm	2D	NA	Hydrogel	Magnetic field
Ref. [5]	2	>1 cm	2D	>1 min	Hydrogel	Temperature
Ref. [6]	2	~100 µm	3D	>1 min	Hydrogel	Temperature
Ref. [7]	2	>1 mm	2D	NA	Hydrogel	Temperature
Ref. [8]	2	~100 µm	3D	~100 ms	Hydrogel	Temperature &light
Ref. [9]	2	>1 cm	2D	>1 s	Hydrogel	Light (175~900 mW)
Ref. [10]	2	<100 µm	2D	<100 ms	Hydrogel	Light (2.5 W)
Ref. [11]	2	<100 µm	3D	300 ms	Hydrogel	Light (70~120 mW)
Ref. [12]	4	>2 cm	2D	>2 s	Liquid crystal elastomer	Different Lights (520, 655, 808 nm, 100~300 Mw)
Ref. [13]	>10	>200 µm	2D	NA	Liquid crystal elastomer	2D structured light (2.5 W)
Ref. [14]	>10	>500 µm	2D to 3D	NA	Shape memory alloy	2D scanning light (188 mW)
This work	>10	<100 µm	3D	30 ms	Hydrogel	3D Structured light (<10 mW)

Supplementary Table 1 Comparison of current soft actuators.

*Modalities numbers: Here we only consider the number of modalities in the steady state.

Supplementary References

1. Sydney Gladman, A., Matsumoto, E.A., Nuzzo, R.G., Mahadevan, L. & Lewis, J.A. Biomimetic 4d printing. *Nat. Mater.* **15**, 413-418 (2016).
2. Li, H., Go, G., Ko, S.Y., Park, J.O. & Park, S. Magnetic actuated pH-responsive hydrogel-based soft micro-robot for targeted drug delivery. *Smart Mater. Struct.* **25**, 027001 (2016).
3. Li, R. et al. Stimuli-responsive actuator fabricated by dynamic asymmetric femtosecond bessel beam for in situ particle and cell manipulation. *ACS Nano* **14**, 5233-5242 (2020).
4. Goudu, S.R. et al. Biodegradable untethered magnetic hydrogel milli-grippers. *Adv. Funct. Mater.* **30**, 2004975 (2020).
5. Pantula, A. et al. Untethered unidirectionally crawling gels driven by asymmetry in contact forces. *Sci. Robot.* **7**, eadd2903 (2022).
6. Nishiguchi, A., Mourran, A., Zhang, H. & Moller, M. In-gel direct laser writing for 3D-designed hydrogel composites that undergo complex self-shaping. *Adv. Sci.* **5**, 1700038 (2018).
7. Malachowski, K. et al. Stimuli-responsive theragrippers for chemomechanical controlled release. *Angew. Chem. Int. Ed.* **53**, 8045-8049 (2014).
8. Hippler, M. et al. Controlling the shape of 3d microstructures by temperature and light. *Nat. Commun.* **10**, 232 (2019).
9. Zhao, Y. et al. Soft phototactic swimmer based on self-sustained hydrogel oscillator. *Sci. Robot.* **4**, eaax7112 (2019).
10. Zhang, H., Mourran, A. & Moller, M. Dynamic switching of helical microgel ribbons. *Nano Lett.* **17**, 2010-2014 (2017).
11. Deng, C.S. et al. Femtosecond laser 4d printing of light-driven intelligent micromachines. *Adv. Funct. Mater.* **33**, 2211473 (2023).

12. Yang, X. et al. Bioinspired light-fueled water-walking soft robots based on liquid crystal network actuators with polymerizable miniaturized gold nanorods. *Nano Today* **43**, 101419 (2022).

13. Palagi, S. et al. Structured light enables biomimetic swimming and versatile locomotion of photoresponsive soft microrobots. *Nat. Mater.* **15**, 647-653 (2016).

14. Han, M. et al. Submillimeter-scale multimaterial terrestrial robots. *Sci. Robot.* **7**, eabn0602 (2022).
