# Supplementary material for: Light-triggered multi-joint microactuator fabricated by two-in-one femtosecond laser writing
Source: Nat Commun. 2023 Jul 17;14:4273. doi: 10.1038/s41467-023-40038-x (PMC10352372; doi:10.1038/s41467-023-40038-x)
Supplement: Supplementary file 3 — Description of Additional Supplementary Files [file 41467_2023_40038_MOESM3_ESM.pdf]

Description of Additional Supplementary Files

Supplementary Movie Legends:

Supplementary Movie 1: Light triggered humanoid microactuator with four joints.

Supplementary Movie 2: Light triggered microplate with short response time (30~60 ms).

Supplementary Movie 3: Light triggered shrinkage of the microplate (100 times).

Supplementary Movie 4: Light triggered micro robotic arm with a joint.

Supplementary Movie 5: Light triggered water strider with four vertical joints.

Supplementary Movie 6: Light triggered humanoid microactuator with multi-joint linkage deformation in XY plane.

Supplementary Movie 7: Light triggered humanoid microactuator with multi-joint linkage deformation in XY and YZ planes.

Supplementary Movie 8: Light triggered micro robotic arm with multijoint collecting multiple microparticles at different locations
